# Spinocerebellar ataxia with mixed tremor and hippocampal atrophy: case report and literature review

**DOI:** 10.3389/fnins.2026.1741827

**Published:** 2026-02-24

**Authors:** Xuan Wang, BingLing Zhou, ZhangBao Guo, Wei Shao

**Affiliations:** 1 The First Clinical Medical School, Hubei University of Chinese Medicine, Wuhan, China; 2 Wuhan Hospital of Traditional Chinese and Western Medicine, Wuhan, China

**Keywords:** ataxia, clinical characteristics, gene, genetics, trinucleotide repeat

## Abstract

**Objective:**

This study aimed to investigate the clinical features of spinocerebellar ataxia 12 (SCA12).

**Methods:**

Sanger sequencing-based genetic testing was performed on a patient initially diagnosed with essential tremor.

**Results:**

The patient exhibited an abnormal expansion of 69 cytosine-adenine-guanine (CAG) repeats, confirming the diagnosis of SCA12.

**Conclusion:**

SCA12 may present with mixed tremor, predominantly postural/kinetic tremor with a superimposed resting component and hippocampal atrophy. However, the direct association between hippocampal atrophy and SCA12 pathology remains unclear and may reflect concomitant age-related or vascular changes. Furthermore, PPP2R2B gene abnormalities may also affect the synergistic function of the basal ganglia-thalamocortical and cerebello-thalamocortical circuits.

## 1 Introduction

Spinocerebellar ataxia type 12 (SCA12) is a late-onset autosomal dominant neurodegenerative disorder. The clinical phenotypic presentation of SCA12 typically includes bilateral upper limb tremor, dysarthria, gait ataxia, hyperreflexia, and neuropsychiatric symptoms (O’Hearn et al., 2015). The age of onset ranges from 8 to 55 years, most often between 40 and 50 years. The prevalence of SCA12 is significantly higher in India compared with other regions (Srivastava et al., 2001; Ganaraja et al., 2022). SCA12 is caused by an unstable expansion of a cytosine-adenine-guanine (CAG) trinucleotide repeat located within the PPP2R2B gene, flanking exon 7 on chromosome 5q32. Affected individuals carry 51–78 CAG repeats (Holmes et al., 1999). Studies have shown that patients carrying 43–50 CAG repeats, when compared with those above the typical pathogenic threshold of 51 repeats, exhibit clinical features similar to the classic SCA12 phenotype but with a highly variable age of onset (Srivastava et al., 2017). This study presents the clinical characteristics and neuroimaging findings of a proband with SCA12, together with a literature review, aiming to improve clinical diagnosis.

A literature search was conducted in the Wanfang, VIP, China National Knowledge Infrastructure (CNKI), and PubMed databases^1^ using the search terms “Spinocerebellar Ataxia-12” and “SCA12” covering publications from 1999 to present. A total of 14 publications reporting affected individuals or pedigrees were identified (Srivastava et al., 2001; Holmes et al., 1999; Srivastava et al., 2017; Ganaraja et al., 2022; Chen et al., 2014; Li, 2011; Li et al., 2025; Xie et al., 2017; Basu et al., 2024; Kalia et al., 2014; Zhao et al., 2002; Hellenbroich et al., 2004; Zheng et al., 2024; Brussino et al., 2010) and are summarized in Table 1.

**Table 1:** Reported cases of SCA12.

Family Report Country	Age of Onset	Clinical Presentation						MRI	SCA12	
		Initial Symptom (Common)	Limb Tremor	Ataxia	Cerebellar Dysfunction (Dysarthria, Nystagmus, etc.)	Extrapyramidal Signs	Psychiatric Symptoms		Normal CAG Range	Pathogenic CAG Expansion
(Holmes et al., 1999) German-American	8–62	Upper limb tremor	√	√	√	√	√	Cerebral and cerebellar atrophy	7–30	66–78
(Srivastava et al., 2001) India	26–50	Bilateral upper limb tremor	√	√	√	√	×	Cerebral and cerebellar atrophy	7–31	55–69
(Zhao et al., 2002) Singapore	This report only presented statistical data on SCA patients in Singapore, including one case of SCA12.									66
(Xie et al., 2017) China	43	Bilateral upper limb tremor	√	√	×	√	×	Cerebellar atrophy	22–27	68
(Hellenbroich et al., 2004) German descent	59	Gait ataxia	√	√	√	×	√	Initially normal; showed bilateral hyperintensities in the lentiform and caudate nuclei, and frontal, parietal, and insular cortices 4 months later	/	49
(Brussino et al., 2010) Italy	45–60	Action tremor in head and hands	√	√	√	√	×	Cerebral and cerebellar atrophy	/	57, 58
(Li, 2011) Chinese Uyghur	28–57	Gait instability, followed or accompanied by upper limb tremor	√	√	√	√	√	Cerebral atrophy was more severe than cerebellar atrophy	7–19	47–53
(Kalia et al., 2014) India	59	Bilateral upper limb tremor and voice tremor	√	√	√	√	√	Mild cerebral and cerebellar atrophy	4–32	67
(Chen et al., 2014) China	40–50	Bilateral upper limb tremor	√	√	√	√	×	Cerebral and cerebellar atrophy	11–17	51–55
(Srivastava et al., 2017) India	18–71	Characteristic action tremor in upper limbs	√	√	√	√	×	Mild to moderate cerebral atrophy, more pronounced than cerebellar atrophy	/	43–73
(Zheng et al., 2024) China	44	Head tremor	√	√	√	√	×	Few ischemic lesions in bilateral lateral ventricles	/	51
(Ganaraja et al., 2022) India	35–57	Bilateral upper limb tremor	√	√	√	√	√	Predominantly cerebellar atrophy, with some cerebral and brainstem atrophy	9–40	43–72
(Basu et al., 2024) North India	58–71	/	√	√	√	√	√	Mild cerebellar atrophy	/	49–56
(Li et al., 2025) China	47–55	Involuntary head tremor	√	√	√	√	√	Severe cerebral cortical atrophy; overall cerebellar hemispheric atrophy but relatively milder	4–32	57, 58

## 2 Case review

### 2.1 The Proband’s first presentation

#### 2.1.1 History and onset

A 71-year-old female patient first noticed involuntary tremors of the head and facial muscles, along with tremors in both upper limbs, in 2010. As the symptoms did not significantly affect her activities of daily living, she did not seek treatment initially. In 2022, she presented to our outpatient clinic for the first time. On physical examination, the patient exhibited tremors of the head and jaw while seated at rest, along with involuntary tremors in both upper limbs when placed on the knees. The tremor was more pronounced on the right side, with an amplitude of approximately 1–2 cm at rest, increasing to 3–4 cm during object holding or voluntary movement. No pill-rolling tremor, bradykinesia, or rigidity was observed. The patient also exhibited changes in speech tone, dizziness, unsteady gait, low mood, and poor sleep.

#### 2.1.2 Auxiliary examinations

**Figure 1:**
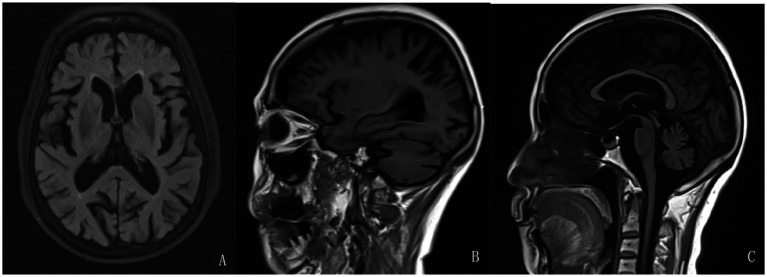
Brain magnetic resonance imaging (MRI) of the patient in 2022. Additionally, **(A–C)**, have shown brain atrophy, ventricular enlargement, and widened, deepened, and enlarged cerebral sulci and fissures.

Magnetic resonance imaging (MRI) findings indicated cerebral atrophy, ventricular enlargement, and widening and deepening of cerebral sulci, fissures, and cisterns, with no midline shift (Figures 1A–C). Neuropsychological assessments indicated cognitive decline, with the following scores: Mini-Mental State Examination (MMSE): 16 points (junior high school level); Montreal Cognitive Assessment (MoCA): 6 points; Hamilton Anxiety Rating Scale (HAMA): 25 points; Hamilton Depression Rating Scale (HAMD): 17 points; and Somatic Self-rating Scale (SSS): 43 points. More extensive neuropsychological testing beyond the MMSE and MoCA was not performed due to limited resources.

#### 2.1.3 Diagnosis and treatment

A provisional diagnosis of essential tremor-plus syndrome (ET-plus) was made. Treatment was initiated with Arotinolol 10 mg twice daily, Trazodone Hydrochloride 50 mg at bedtime, Flupentixol–Melitracen one tablet daily, Eszopiclone 2 mg at bedtime, and Citalopram Hydrobromide 20 mg once daily. On follow-up, her dizziness improved; however, the tremors remained largely unchanged.

### 2.2 Proband’s second presentation

#### 2.2.1 Symptom evolution

On 31 May, 2024, the patient returned to the clinic. She reported worsening of her long-anding bilateral hand tremor and dizziness, along with new-onset fatigue lasting 3 days. The tremors persisted. In addition, she exhibited significant cerebellar ataxia, presenting with dizziness, a sense of head heaviness, lightheadedness, and gait imbalance described as “walking on cotton.” The patient also exhibited occasional diplopia (double vision), indicating oculomotor dysfunction, and hoarseness and choking while drinking, indicating bulbar dysfunction. Autonomic symptoms were present as constipation, and symptoms of sleep and psychological disorders included irritability, quick temper, sleep disturbances, without nocturnal screaming, punching, or kicking behaviors. Other neurological symptoms/signs included distending pain in the vertex and bilateral temporal regions (VAS 3–5) and a sensation of neck stiffness. The patient’s medical history included cerebral infarction and atherosclerosis, with long-term oral aspirin use and Naodesheng concentrated pills. She also had a history of bilateral upper limb fractures 8 years earlier and essential tremor for over 10 years, which had been previously treated with arotinolol (Almarl) without any effect and was subsequently discontinued. The patient’s family history had indicated that both parents are deceased; her father had a history of tremor but did receive no specific treatment. The patient has six siblings, with multiple family members experiencing tremor symptoms, none of whom have received specific treatment..

#### 2.2.2 Physical examination

The patient was alert and oriented. She presented with dysarthria, hoarseness, and a festinating gait. Cranial nerve examination was unremarkable. On examination, muscle strength was graded 5 in all four limbs, with increased tone. She exhibited vertical nystagmus, bilateral inaccuracy on the finger-to-nose test, and clumsy rapid alternating movements, and she was uncooperative for the Romberg test. Functional activities such as eating, combing hair, buttoning, writing, and walking were impaired due to tremor and ataxia. The water drinking test (the Wada test) was grade 2. Tendon reflexes, autonomic nervous system, and pathological signs were all normal. Notably, standardized motor scales, such as the Unified Parkinson’s Disease Rating Scale part III (UPDRS-III) and the Scale for the Assessment and Rating of Ataxia (SARA), were not employed for quantitative assessment during the clinical visit.

#### 2.2.3 Laboratory investigations

Complete blood count revealed a hemoglobin level of 107 g/L and a hematocrit of 33.5%. Liver function tests showed a total serum protein level of 64.2 g/L and a serum albumin level of 38.7 g/L. Electrolyte analysis revealed a serum calcium level of 2.18 mmol/L. Lipid profile included triglycerides of 1.71 mmol/L, non-high-density lipoprotein cholesterol of 4.27 mmol/L, and low-density lipoprotein cholesterol of 3.58 mmol/L. Coagulation profile, plasma D-dimer assay, folate level, serum vitamin B12 assay, renal function, cardiac enzyme profile, plasma homocysteine level, fecal occult blood test (immunochemical method), and glucose assays (various enzymatic methods) were all essentially within normal limits.

**Figure 2:**
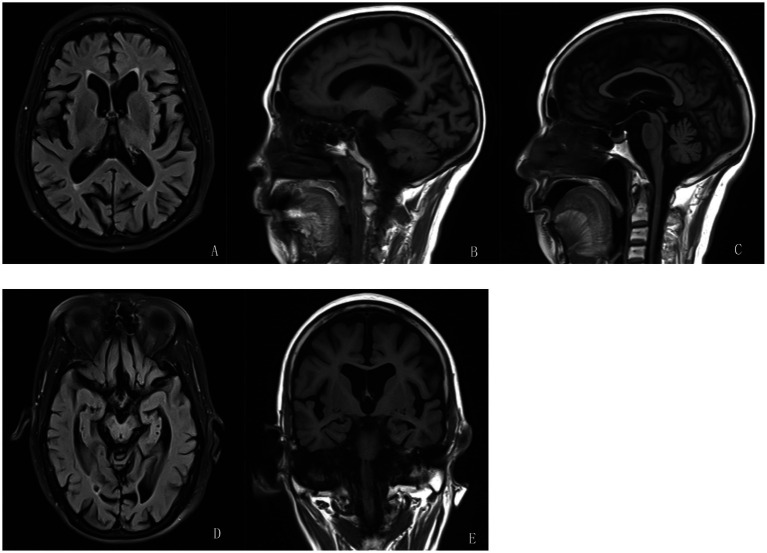
Brain magnetic resonance imaging (MRI) of the patient in 2024. Additionally, **(A)** Brainstem degeneration and/or ischemia; lacunar infarctions in the bilateral parietal lobes and the right semioval center. **(B,** **C)** Cerebellar atrophy. **(D,** **E)** Small, round-like lesions exhibiting long T1 and long T2 signals were observed in the bilateral hippocampi.

Cranial MRI indicated: brainstem degeneration and/or ischemia; lacunar infarctions in the bilateral parietal lobes and the right semioval center (Figure 2A); cerebellar atrophy (Figures 2B,C); and hippocampal atrophy (bilateral reduction in hippocampal volume with enlargement of the choroidal fissures and temporal horns of the lateral ventricles). Small, round-like lesions exhibiting long T1 and long T2 signals were observed in the bilateral hippocampi (Figures 2D,E).

**Figure 3:**
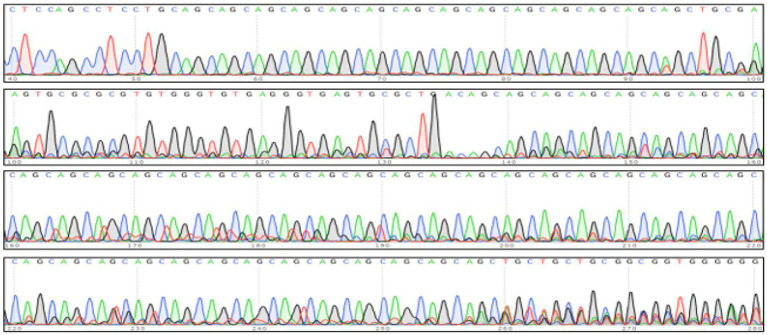
Genetic testing graph.

Based on the patient’s presentation of mixed tremor (predominantly in the right upper limb), imaging findings (hippocampal atrophy), and a family history of tremor, genetic testing for SCA subtypes was performed. Sanger sequencing revealed approximately 69 CAG repeats (normal threshold ≤43) in one allele of the PPP2R2B gene, supporting a diagnosis of SCA12 (Figure 3).

#### 2.2.4 Treatment and prognosis

Symptomatic treatment included neurotrophic agents, antivertigo medication, sleep improvement, and mood regulation. The patient’s dizziness fluctuated in severity, while involuntary tremors persisted in both upper limbs (which was more pronounced on the right), accompanied with head and jaw tremors. During follow-up, the symptoms remained stable without improvement, but no significant worsening has been observed to date.

Primers were designed to target the flanking sequences on both ends of the trinucleotide repeat region of the target (PPP2R2B) gene. The repeat sequence was amplified using polymerase chain reaction (PCR), and the PCR products were then subjected to Sanger sequencing. An analysis revealed that the calculated number of (CAG) n repeats in the PPP2R2B gene on one chromosome was approximately 69.

### 2.3 Family investigation

**Figure 4:**
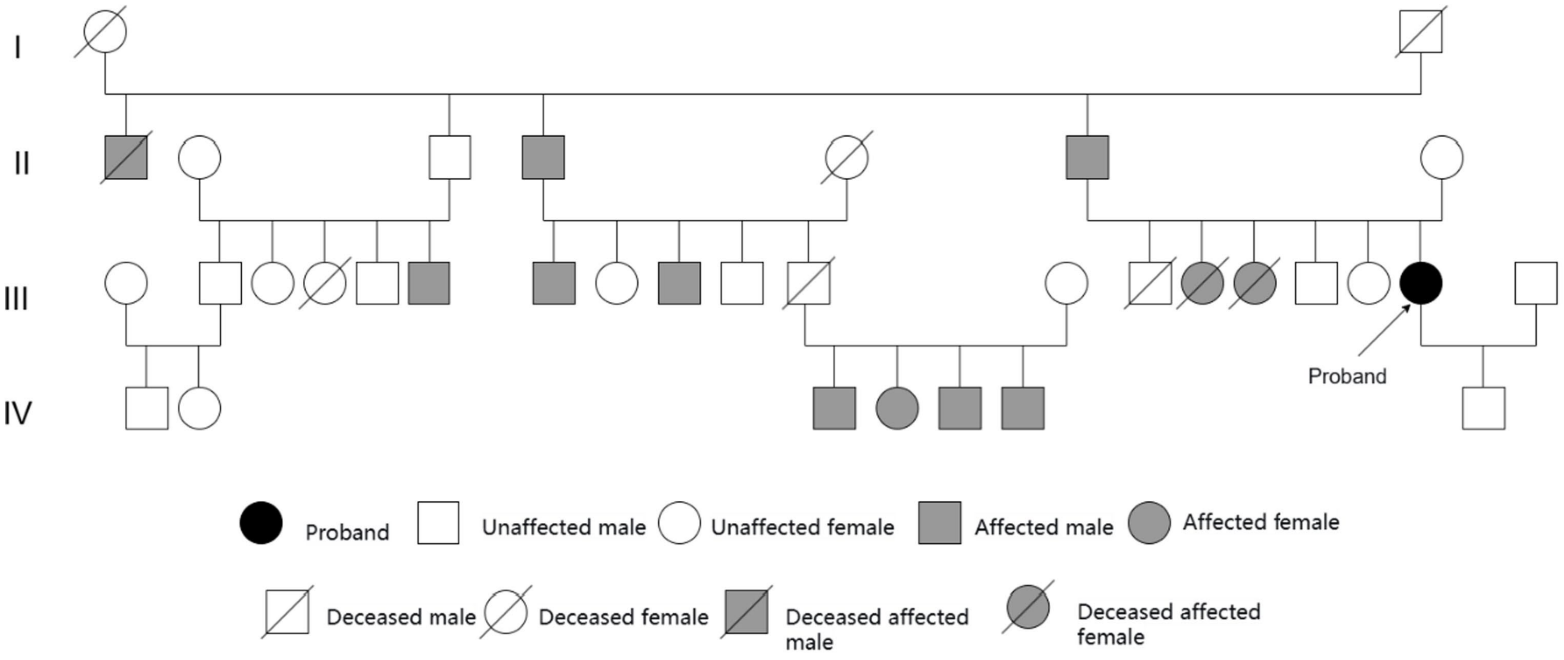
Pedigree chart of the SCA12 proband.

Figure 4 depicts the pedigree analysis. The family pedigree spans four generations and includes 36 members, of whom 14 exhibited tremor symptoms. No tremor was reported in the first generation. In the second generation, three individuals were affected, while seven individuals were affected in the third generation. However, due to factors such as geographical dispersion, advanced age, and financial constraints, genetic testing or detailed neurological assessments could not be performed in these symptomatic relatives, limiting our analysis of the hereditary characteristics of SCA12 in this family. In the fourth generation, four individuals exhibited tremor symptoms. Among them, four members, including the proband’s son, underwent SCA12 genetic testing, and all of their results were negative.

## 3 Literature review and discussion

A review of the 14 publications revealed that SCA12 was first reported and named by Holmes et al. in 1999 (Holmes et al., 1999). Typical clinical manifestations of SCA12 include limb tremor, ataxia, and cerebellar dysfunction. In contrast, this case was initially presented with head and jaw tremor, accompanied by mixed tremor in both upper limbs. Kinetic tremor of the upper limbs is more commonly observed at disease onset in SA12. Although resting tremor was observed, its marked exacerbation during postural maintenance and intentional movement aligns more with a mixed tremor—primarily postural/kinetic with a superimposed resting component—rather than a classic Parkinsonian-type resting tremor. One explanation is that tremor generation is related to abnormal activity in the cerebellar–thalamic–cortical pathway. The cerebellum integrates both the sensory and motor information from the cortex and modulates the motor cortex via thalamic feedback (Li et al., 2023). Within the cerebellar–thalamic–cortical circuit, the motor cortex plays a specific role in determining tremor amplitude, acting as a “dimmer switch” to regulate its intensity (Malling et al., 2019). The inhibitory output from the basal ganglia, particularly the internal segment of the globus pallidus, exerts a crucial ‘switch’-like control over thalamic activity (Helmich et al., 2012). Motor commands are relayed to the internal globus pallidus of the basal ganglia, which then sends inhibitory signals to the thalamus, thereby regulating the motor cortex excitability and downstream corticospinal tract activity, ultimately initiating muscle contraction and movement (Jellinger, 2019).

The PPP2R2B gene encodes the Bβ regulatory subunit of protein phosphatase 2A (PP2A). By modulating PP2A activity toward substrates such as the microtubule-associated protein Tau, this subunit participates in tau dephosphorylation (Lin et al., 2010). Dysfunction in this process may lead to tau detachment from microtubules, its subsequent aggregation into neurofibrillary tangles (NFTs), and ultimately the disruption of the neuronal cytoskeleton. This impairs axonal transport and synaptic function, culminating in neuronal death (Chen et al., 2009). PPP2R2B dysfunction may lead to dysregulation of PP2A activity toward pathways such as MAPK/ERK, impairing neuronal growth, differentiation, survival, and synaptic function. Such dysfunction has been linked to intellectual disability and developmental delay, and represents a potential mechanism underlying spinocerebellar ataxia (Sandal et al., 2025). PP2A also plays a role in dopaminergic signaling, and dysfunction of the dopaminergic system is closely associated with movement disorders such as tremor (Rampino et al., 2017). Pathologically, PPP2R2B abnormalities predominantly affect the cerebellum and the cerebral cortex, manifesting as Purkinje cell degeneration and cortical neuronal loss (Cohen and Margolis, 2016). Based on the clinical phenotype observed in this patient, the mixed tremor suggests that PPP2R2B gene abnormalities may impair the functional synergy between the basal ganglia–thalamocortical and cerebellar–thalamocortical circuits. Previous reports have not commented on tremor asymmetry; therefore, it remains unclear whether the more pronounced right-sided tremor observed in this patient is typical. A comparison of tremor characteristics in SCA12 between previously reported cases and the present case is provided in Table 2.

**Table 2:** Comparison of tremor characteristics in SCA12.

Source	Tremor Characteristics	Resting Component Present
(Holmes et al., 1999) German–American	Upper extremity tremor	×
(Srivastava et al., 2001) India	hand tremor	×
(Zhao et al., 2002) Singapore	/	×
(Xie et al., 2017) China	Intentional tremor	×
(Hellenbroich et al., 2004) German escent	A striking action tremor	×
(Brussino et al., 2010) Italy	Action tremor of the upper extremities	×
(Li, 2011) Chinese Uyghur	Bilateral upper limb tremor	×
(Kalia et al., 2014) India	4-Hz postural tremor	×
(Chen et al., 2014) China	Bilateral upper limb tremor	×
(Srivastava et al., 2017) India	Asymmetric onset of tremor in the right hand	×
(Zheng et al., 2024) China	Kinetic and postural tremor	×
(Ganaraja et al., 2022) India	Postural/intentional tremor	×
(Basu et al., 2024) North India	Postural and action tremors	×
(Li et al., 2025) China	Tremor of the right-hand-held object	×
Present case	Mixed tremor (postural + resting)	√

Extrapyramidal symptoms such as increased muscle tone, psychiatric symptoms including dementia and depression, and Parkinsonian symptoms such as bradykinesia and rigidity, often do not appear at the onset or in the early stages of SCA12. Cognitive impairment may typically emerge as the disease progresses. The progressive neurodegeneration in SCA12 gradually affects both cortical and subcortical regions (Basu et al., 2024) but shows no correlation with age, age of onset, ataxia severity, disease duration, or CAG repeat length (Agarwal et al., 2021).

## 4 Differential diagnosis

In this pedigree, the proband presented with a rare initial symptom of head, facial, and jaw tremor, followed by tremors in both upper limbs, which were also observable at rest. These symptoms were accompanied by ataxia, dysphagia, dysarthria, and cognitive impairment. In the early stages of the disease, other SCA12 patients have been diagnosed with essential tremor (ET) (O’Hearn et al., 2001). However, in this pedigree, the proband was initially diagnosed with ET-plus. The ET-plus refers to the presence of characteristic bilateral upper limb action tremor of ET, along with additional neurological signs of uncertain significance, such as impaired tandem gait, questionable dystonic posturing, memory impairment, or other mild neurological signs of unclear significance, while excluding dystonia and task-specific tremor (Zhang, 2023). Symptomatic treatment proved ineffective, and SCA12 was not initially considered.

In the elderly population, multiple neurodegenerative disorders may coexist, leading to complex clinical presentations. For example, neurodegenerative diseases such as Parkinson’s disease (PD) or Alzheimer’s disease (AD) can occur alongside cerebrovascular diseases (e.g., cerebral infarction, leukoencephalopathy), representing a mixed pathology (Narasimhan et al., 2022). In such contexts, tremor manifestations can vary and may include resting tremor (typical of PD), postural tremor, or intention tremor, which was more commonly linked to cerebellar lesions or essential tremor (Jellinger, 2025). This necessitates a comprehensive evaluation integrating clinical, imaging, neurophysiological, and genetic information. Although the patient had cerebrovascular risk factors, gait disturbance, and resting tremor—features requiring differentiation from vascular parkinsonism (VaP)—the typical clinical presentation of VaP is often characterized as a “lower-body parkinsonism” or “parkinsonism of the lower half”. The VaP involves rigidity and bradykinesia in both lower limbs and postural instability of the trunk, while upper limb function generally remains intact (Wang, 2022). However, these patterns does not align with the symptoms observed in the present case. After several medical consultations, further inquiry into the family history revealed that several family members had limb tremors. Genetic testing was recommended for the patient, which detected 69 CAG repeats, confirming the diagnosis of SCA12. However, the possibility that cerebrovascular disease contributed to certain clinical features, such as gait instability and some cognitive impairment, cannot be entirely excluded.

When symptoms such as resting tremor, ataxia, and hippocampal atrophy are present in a patient with a significant family history, SCA12 should be considered. Differentiating SCA12 from essential tremor can be clinically challenging;in such cases, eliciting a detailed family history is crucial. Additionally, epidemiological clues, such as Indian ancestry, may also raise suspicion for SCA12 (Kalia et al., 2014). Genetic counseling should be provided to family members regarding autosomal dominant cerebellar ataxia (ADCA).

In patients with SCA12, structural MRI primarily reveals atrophy of the cerebral cortex and the cerebellum. The degree of cerebral cortical atrophy is generally more severe than that of the cerebellar cortex (Holmes et al., 1999; Chen et al., 2014), with the vermis more prominently affected than the cerebellar hemispheres. Atrophy rarely involves the basal ganglia, thalamus, brainstem nuclei, and subcortical regions of the cerebrum (Li et al., 2012; Yao et al., 2022). In this case, the proband exhibited more pronounced cerebral cortical atrophy compared to cerebellar hemispheric atrophy, consistent with some reports related to SCA12. Although hippocampal atrophy was observed—particularly noteworthy given the family history and tremor—and raised suspicion for a hereditary degenerative ataxia such as SCA12, it should be noted that hippocampal atrophy is a common imaging finding in the elderly population. This study lacked quantitative volumetric measurements or comparison with an age-matched control group. Therefore, we cannot determine whether this represents a specific neuroimaging marker of SCA12 or reflects age-related or vascular changes.

In clinical practice, the presence of mixed tremor, characterized by postural/kinetic and resting components, alongside hippocampal atrophy, particularly in the context of a positive family history, should prompt consideration of SCA12. Early genetic testing enables accurate diagnosis and prevents misdiagnosis as ET or other neurodegenerative disorders.

## 5 Treatment

No specific targeted therapy for SCA12 has been identified to date. According to international reports (Ganaraja et al., 2022), drug treatments for SCA12 patients often involve a combination therapy with agents such as amantadine, propranolol, clonazepam, primidone, levodopa, trihexyphenidyl, and baclofen. However, no objective evaluations of these treatments are available. An earlier study on the use of propranolol for SCA12 showed no significant improvement in tremor symptoms (O’Hearn et al., 2001). In contrast, a case report of an SCA2 patient with severe action tremor demonstrated marked symptom improvement following combined deep-brain stimulation (DBS) of the thalamus and subthalamic nucleus (Freund et al., 2007). In the present case, the proband showed minimal symptomatic change after treatment with the antitremor agent Arotinolol.

## 6 Summary

In summary, this familial case and literature review reaffirm that upper limb tremor is the most frequent initial symptom in SCA12, often accompanied with head and voice tremors. This case exhibits that SCA12 can manifest with a mixed tremor phenotype—prominent postural/intentional tremor with a significant resting component—together with pyramidal and extrapyramidal signs and cognitive impairment. Notably, hippocampal atrophy was observed, whether this finding is specific to SCA12 or relates to aging or vascular changes remains unclear and requires further investigation.

This case report is limited to a single family, which constrains the generalizability of the findings. Furthermore, the available imaging and genetic data for the family are incomplete. There is also a lack of documentation regarding early symptoms and signs, as well as early-stage and familial neuroimaging data. Importantly, definitive evidence of familial co-segregation is absent, as genetic testing was not performed on other symptomatic relatives, preventing confirmation of the phenotype–genotype link within the family.

Therefore, in clinical practice, it is essential to develop a systematic diagnostic approach for tremor by obtaining detailed patient histories, emphasizing neuroimaging evaluation, exploring functional imaging assessments, and applying genetic testing selectively. Future prospective multicenter cohort studies with large sample sizes are warranted for SCA12. The use of quantitative MRI techniques, such as volumetric measurement and diffusion tensor imaging, combined with refined neuropsychological testing batteries, may help determine the specific hippocampal involvement in SCA12.

## Data Availability

The datasets presented in this study can be found in online repositories. The names of the repository/repositories and accession number(s) can be found in the article/supplementary material.

## Footnotes

1
http://www.ncbi.nlm.nih.gov/pubmed
