# Supplementary figures and images for: Spinocerebellar ataxia with mixed tremor and hippocampal atrophy: case report and literature review
Source: Front Neurosci. 2026 Feb 24;20:1741827. doi: 10.3389/fnins.2026.1741827 (PMC12971692; doi:10.3389/fnins.2026.1741827)

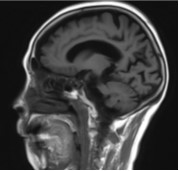

Supplement: Supplementary file 1 [file Image_1.JPEG]

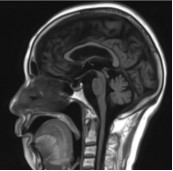

Supplement: Supplementary file 2 [file Image_2.JPEG]

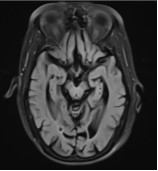

Supplement: Supplementary file 3 [file Image_3.JPEG]

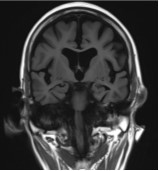

Supplement: Supplementary file 4 [file Image_4.JPEG]

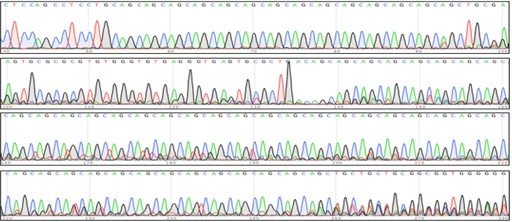

Supplement: Supplementary file 5 [file Image_5.JPEG]

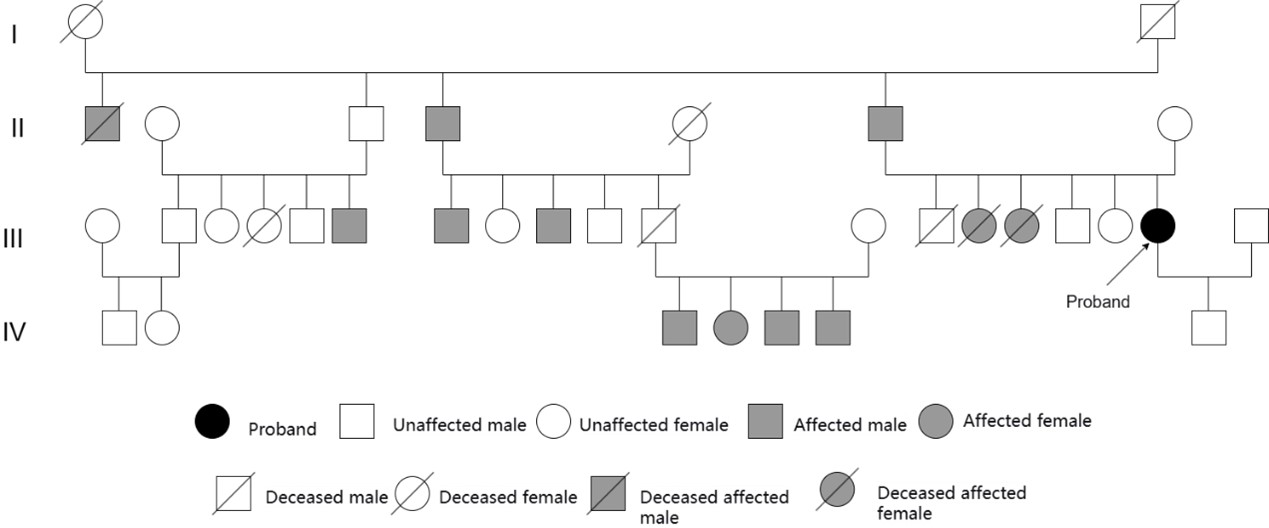

Supplement: Supplementary file 6 [file Image_6.JPEG]
